# Socially Anxious Individuals Get a Second Chance After Being Disliked at First Sight: The Role of Self-Disclosure in the Development of Likeability in Sequential Social Contact

**DOI:** 10.1007/s10608-012-9449-4

**Published:** 2012-03-29

**Authors:** M. J. Voncken, K. F. L. Dijk

**Affiliations:** 1 Department of Clinical Psychological Science, Maastricht University, P.O. box 616, 6200 MD Maastricht, The Netherlands; 2 Department of Clinical Psychology, University of Amsterdam, Amsterdam, The Netherlands

**Keywords:** Social anxiety, Social anxiety disorder, First impression, Likeability, Social interaction, Self-disclosure

## Abstract

Socially anxious individuals (SAs) not only fear social rejection, accumulating studies show that SAs are indeed judged as less likeable after social interaction with others. This study investigates if SAs already make a more negative impression on others in the very first seconds of contact. The study further investigates the development of likeability and the role of self-disclosure herein in two sequential social interactions: first after an unstructured waiting room situation and next after a ‘getting acquainted’ conversation. Results showed that high SAs (n = 24) elicited a more negative first impression than low SAs (n = 22). Also, although high SAs improved from the first to the second task, they were rated as less likeable after both interactions. The level of self-disclosure behaviour was the strongest predictor for the development of likeability during the sequential social tasks. The absence of an interaction between group and self-disclosure in predicting the development of likeability suggests that this is true for both groups. Thus, high SAs can improve their negative first impression if they are able to increase their self-disclosure behaviour. However, SAs showed a decreased level of self-disclosure behaviour during both social interactions. Targeting self-disclosure behaviour may improve the negative impression SAs elicit in others.

## Introduction

Patients with Social Anxiety Disorder (SAD) are afraid that others do not like them. Cognitive models of SAD (Clark 2005; Hofmann 2007; Rapee and Heimberg 1997) argue that a biased perception of how others view them is central to SAD. However, an increasing number of studies demonstrated that social anxiety is indeed related to social rejection. That is, people with social anxiety are more negatively evaluated than people without social anxiety (Alden and Wallace 1995; Creed and Funder 1998; Heerey and Kring 2007; Meleshko and Alden 1993; Pilkonis 1977; Voncken et al. 2008). Furthermore, there is also evidence that socially anxious adolescents are treated more negatively by their peers than their non-socially anxious classmates (Blöte et al. 2007). Taken together, the concern of socially anxious individuals to be less liked seems partly valid and could fuel their social anxiety. Interpersonal models of SAD (Alden 2001), therefore, put forward that their problems of being seen as less likeable might be a crucial maintenance problem in SAD.

Although actual likeability problems are proposed to be critical in the maintenance of SAD, there is little known about the impression socially anxious individuals evoke in the first seconds of social contact. From research into such very first impressions, it is known that participants make stable judgments about personal characteristics such as attractiveness and likeability, already after 100 ms exposure to a face (Willis and Todorov 2006). Although evidence is available that socially anxious individuals are liked less after getting acquainted interactions of 5–10 min (Alden and Wallace 1995; Creed and Funder 1998; Meleshko and Alden 1993; Voncken et al. 2008), it might be that they, already in the very first seconds, elicit negative responses from others.

In addition, it would be of interest to investigate how the judgments others have of people with social anxiety develop after such a first impression. For instance, Wilder and Thompson (1980) showed that people form more favourable views toward whomever they spent time with, even if these others are members of a previous disliked or stereo-typed out-group. However, this study was conducted with healthy participants. It can be hypothesized that, in contrast, socially anxious individuals elicit even more negative responses in others than their non-socially anxious counterparts after prolonged social interaction. That is, cognitive models (Clark 2005) put forward that SAD patients tend to use safety behaviours in order to prevent a negative impression on others. These strategies have been assumed to disturb social interaction (McManus et al. (2008).

It would be important to gain insight in the specific behaviour that contributes to the likeability of people with social anxiety. For example, the mutual increase of self-disclosure is viewed to be fundamental to likeability and the development of social relationships (Altman and Taylor 1973; Jourard 1971). Self-disclosure refers to how open one is about oneself to another person (Collins and Miller 1994). Studies investigating self-disclosure behaviour indicate the so-called *liking effect*: people who naturally self-disclose or are instructed to self-disclose are more liked than people who do not (see for a review Collins and Miller 1994). To date, only two studies investigated the role of self-disclosure in social anxiety and its effect on how likeable these individuals were rated by others. Alden and Bieling (1998) were able to show that socially anxious individuals do have difficulties with self-disclosure and the reciprocity of self-disclosure. However, in the study of Papsdorf and Alden (1998) the relation between social anxiety and level of self-disclosure was, although significant, rather small (r = 0.27). Both these studies used global self-disclosure ratings of the participants’ performance. The assessment of more detailed and specific self-disclosure behaviour might help to find stronger relations with social anxiety. For instance, Dalto and Ajzen (1979) showed that people revealing positive information are liked more than people revealing negative information. Moreover, people tend to like persons to whom they disclose (see review in Collins and Miller 1994). Therefore, someone’s ability to elicit self-disclosures of other people might be an important factor in being perceived as likeable as well. Lastly, the results of Berg and Archer (1980) suggest that how empathic a person handles disclosures of their interaction partners are also of importance for the development of likeability. That is, characters in a script that made statements of acknowledgement or sympathy in response to a self-disclosure of their interaction partners were favoured by participants over characters that only disclosed personal information in response to a self-disclosure (i.e., reciprocal self-disclosure). Therefore, we chose to assess these specific self-disclosure behaviours more detailed in the current study.

Furthermore, the setting in which the participants are observed is of importance. Most social behaviour observation studies in the social anxiety field used structured social interactions in which participants received instructions about the upcoming social interaction (e.g., getting to know your conversation partner) and participants were aware that they were observed by a video camera. Only one study did include an unstructured, natural social interaction next to a structured social interaction (Thompson and Rapee 2002). In this study participants were unaware that they were video-taped while waiting together with a confederate for the research assistant to return to start the structured social interaction. This study demonstrated that behavioural problems of socially anxious individuals are even more pronounced in this unstructured social interaction compared to a structured interaction. It might be that confronted with a clear social task in which they cannot avoid interacting and disclosing at least some things about themselves, as in the structured setting, socially anxious individuals perform better than in an unstructured social situation. Therefore, in the assessment of the development of likeability in social anxiety it is important to include an unstructured social task next to a structured social interaction.

This study aimed to investigate the very first impression of socially anxious individuals and the development of likeability from a naturalistic, unstructured to a structured social interaction. Furthermore, it was explored whether self-disclosure related behaviours, positive and negative self-disclosures and eliciting and handling self-disclosures of one’s interaction partner, related to the development of likeability. For reasons of convenience and homogeneity of the sample, it was chosen to only include females. Female students with high and low social anxiety were asked to participate in a videotaped 5 min ‘getting acquainted’ conversation with a male confederate. Before this conversation they waited for 5 min in a ‘waiting room’ with a hidden camera. Here the confederate they would meet during the conversation was seated. The confederate rated his first impression and rated the participant on likeability after both the waiting room and getting acquainted conversation. Video-raters rated their first impression, likeability and four difference self-disclosure behaviours of the participants during both interactions.

It was hypothesized that (I) the high socially anxious individuals (SAs) compared to the low SAs group had a more negative first impression (i.e., first few seconds) and were rated as less likeable after both the waiting room and the social interaction. (II) It was expected the high SAs would show less self-disclosure behaviour across both tasks and that this differences between the groups would be most pronounced in the waiting room situation compared to the getting-acquainted task. Last, (III) the increase in likeability during the waiting room and during the getting-acquainted task was expected to be predicted by negatively by group and positively by the level of self-disclosure.

## Method

### Participants

During a pre-screening, 229 first year female Dutch speaking students of psychology and health sciences filled out the Social Interaction Anxiety Scale (SIAS; Mattick and Clarke 1998). First, students (n = 1) that scored 1.5 *SD* above the mean of the SIAS of patients with SAD (as reported by Mattick and Clarke 1998) mean SIAS: 34.6; SD = 16.4: e.g., equal or above 60), and students (n = 9) who scored 1.5 *SD* below the mean of the SIAS in the current study (e.g., equal or lower than 6 on the SIAS; mean SIAS = 23.6, SD = 12.3), were excluded as these may be a deviant levels of SA (see for instance Hofmann and DiBartolo (2010), who state that too little social anxiety is dysfunctional). To compose the high SAs group the top 25% of the individuals with the highest score (n = 60, range SIAS 30–60) were invited for participation and 25% of the students with the lowest scores (n = 58, range SIAS 7–15) were invited.

Of the selected individuals (n = 118) 61 individuals participated. The other 57 individuals did not respond to our mails or voice-mail messages. No differences were found between the individuals that did and did not participate for both the high, t (58) = 1.1, *ns,* and low, t (56) = 0.3, *ns*, SAs group. Because the pre-screening of the SIAS occurred during a mass screening several months before the experiment we carefully checked whether the selected participants could still be considered as high or low SAs. Therefore, the participants filled-out the SIAS again at the assessment of our study. With a median spilt we divided our sample again into a high and low SAs group. The final high SAs group was composed only of individuals that consistently, at both assessment points, were assigned to the high SAs group and the low SAs group of individuals consistently assigned to the low SAs group. This resulted in the exclusion of 10 participants. That is, 6 participants in the low SAs group had higher SIAS scores than the median SIAS score and 4 participants in the high SAs group showed the reverse pattern. These individuals were excluded from the analyses. Finally, 24 high SAs and 25 low SAs females were included in the analyses. The groups did not differ in age (high SAs: M = 19.5, SD = 1.6; low SAs: M = 19.1, SD = 1.4, t(44) = 0.9, *p* > 0.10). Video material of 1 high and 3 low SAs participants was lost due to technical problems.

### Questionnaires

#### Social Anxiety and Depression

The Social Interaction Anxiety Scale (SIAS; Mattick and Clarke 1998) was used to assess the level of social anxiety. The SIAS consists of 20 items that are rated from 0 (not at all characteristic or true of me) to 4 (extremely characteristic or true of me). Items are self-statements describing one’s typical cognitive, affective, or behavioural reactions to situations that involve social interaction in dyads or groups. The SIAS is scored by summing the ratings (after reversing the 3 positively-worded items), and total scores range from 0 to 80. Higher scores represent higher levels of social interaction anxiety. Mattick and Clarke (1998) demonstrated the reliability and validity of the SIAS. This Dutch translation of the SIAS has not been validated. Nevertheless, it is often used in studies and these showed that its internal consistency is good and that it was able to distinguish high fearful individuals from low fearful individuals (e.g., Voncken et al. 2010; Heinrichs et al. 2006). Alfa in the present sample was 0.95. For a more comprehensive description of the sample, the participants also completed the Beck Depression Inventory (BDI, Beck et al. 1961) to assess the level of depressive symptoms. The Dutch translation of the BDI proved to show high internal consistency and, the validity index satisfies general psychometric criteria (van der Does 2002). Alpha in the present sample was 0.88. As expected the high SAs group had higher ratings on the SIAS and the BDI than the low SAs group (SIAS: high SAs: M = 40.0, SD = 12.6; low SAs: M = 15.4, SD = 4.9, t(47) = 9.1, *p* < 0.001; BDI: high SAs: M = 11.8, SD = 7.2; low SAs: M = 4.2, SD = 4.0, t(47) = 4.5, *p* < 0.001).

### First Impression

The first impression of the participants was rated by the confederate and the video raters (see also the procedure section). For the first impression we needed a very rough scale that confederates and video raters could use without much cognitive effort because first impressions can be fast and unreflective (Willis and Todorov 2006). In the Netherlands people often judge the valence of people or attributes with the education grading system. Therefore, each participant was rated by the confederates as well as by the video raters with the Dutch education grading system. The Dutch grading system consists of a scale from 1 (highly insufficient) to 10 (highly sufficient). For passing exams a ‘5’ is insufficient, a ‘6’ is only just sufficient, a ‘7’ is rather well, a ‘8’ is very well, a ‘9’ is excellent and a ‘10’ is without any mistakes and hardly possible to obtain. The Intraclass Correlation Coefficient (two way mixed model and absolute agreement) showed a good interrater reliability for this measure (ICC = 0.80).

### Likeability Ratings (Desire for Future Interaction, DFI)

Likeability of the participants was rated by the confederate and the video raters with the desire for future interaction scale (DFI; Coyne 1976). The DFI comprises eight items rated on 5-point Likert-type scales that measure the extent to which the rater wishes to engage in future social activities with the participant (sample items: ‘Would you like to spend time with the participant?’; ‘Would you like to share a 3-h bus ride with the participant?’; ‘Would you invite the participant to visit you?’). The DFI is a well-established questionnaire that is generally interpreted as a measure of social rejection or liking. It has been shown to be highly reliable, also in Dutch speaking samples (e.g. Boswell and Murray 1981; Papsdorf and Alden 1998; Voncken et al. 2008; Voncken et al. 2010; Winer et al. 1981). Intraclass Correlation Coefficients (two way mixed model and absolute agreement) were used to inspect the interrater reliability in the current study. The ICC between all raters was moderate to good (DFI waiting room: 0.80; DFI getting-acquainted task: 0.67). Internal consistency in the present sample was good, all Alfa’s were >0.90. For the participants (1 high and 3 low SAs) of whom video material was lost the first impression and DFI ratings of the confederates were used.

### Self-Disclosure Behaviour Scale

The Self-disclosure Behaviour Scale was composed out of four scales which were rated by all three video-raters on a 9-point Likert scale: (1) positive self-disclosure (e.g., “How much did you get to know about the participant concerning superficial but positive topics, “To what extent did the participant show positive emotions?”) (2) negative self-disclosure (e.g., “How much did the participant tell about superficial but negative topics”, “In what extent did the participant share about things she had difficulties with”, “To what extent did the participant show negative emotions”); (3) eliciting self-disclosure in the confederate (e.g., “When the participant asked questions: to what extent did the participant show genuine interest when she asked the confederate questions”); (4) responding to self-disclosure of the confederate (e.g., “How does the participants handles information the confederate displays: to what extent does the participant genuinely listens to the confederate when he displays information”, “How empathic did the participant respond to information the confederate displayed?”).

See Table 1 for the Cronbach’s alphas and Intraclass Correlation Coefficients of the subscales of the Self-disclosure Behaviour Scale and Table 2 for correlations between these subscales. The consistency and interrater reliability of the positive self-disclosure, eliciting self-disclosure and responding to self-disclosure was good and the correlations between these three subscales were high. However, for the negative self-disclosure the consistency was only low to good and the interrater reliability for the getting-acquainted task was low (see Table 1). In addition, the correlations of the negative self-disclosure scale with the others scales were moderate in the waiting room and low in the getting-acquainted task. Inspecting the means of the negative self-disclosure scale revealed that the video-raters observed only few negative self-disclosures in the social tasks (waiting room M = 2.6; SD = 1.5; getting-acquainted task: M = 4.0; SD = 1.1). This seems to indicate that negative self-disclosure did not occur with enough frequency to gain reliable ratings for this subscale. Therefore, this subscale was excluded from further analyses. One self-disclosure behaviour mean score was then constructed from the three reliable subscales: positive self-disclosure, eliciting self-disclosure and responding to self-disclosure.

**Table 1:** Cronbach’s alphas for each video-rater and Intraclass correlation coefficient (ICC) for each of the subscales of the self-disclosure behaviour scale

Waiting room					Getting-acquainted task				
Subscales self-disclosure behaviour scale	Cronbach’s alpha			ICC	Subscales self-disclosure behaviour scale	Cronbach’s alpha			ICC
	Rater 1	Rater 2	Rater 3			Rater 1	Rater 2	Rater 3	
Positive self-disclosure	0.94	0.95	0.93	0.93	Positive self-disclosure	0.83	0.91	0.83	0.86
Negative self-disclosure	0.71	0.87	0.74	0.85	Negative self-disclosure	0.61	0.79	0.29	0.57
Eliciting self-disclosure	0.92	0.99	0.98	0.86	Eliciting self-disclosure	0.90	0.99	0.86	0.78
Responding to self-disclosure	0.93	0.98	0.90	0.92	Responding to self-disclosure	0.88	0.94	0.83	0.82

**Table 2:** Simple correlations of the mean subscales of the self-disclosure behaviour scale rated by the three video-raters during the waiting room situation and getting-acquainted task

Waiting room				Getting-acquainted task			
Subscales self-disclosure behaviour scale	2.	3.	4.	Subscales self-disclosure behaviour scale	2.	3.	4.
1. Positive self-disclosure	0.52*	0.87*	0.92*	1. Positive self-disclosure	0.09	0.62*	0.59*
2. Negative self-disclosure		0.41*	0.43*	2. Negative self-disclosure		−0.11	−0.21
3. Eliciting self-disclosure			0.95*	3. Eliciting self-disclosure			0.94*
4. Responding to self-disclosure				4. Responding to self-disclosure			

*Correlation is significant at the 0.01 level

### Confederates

Five male confederates (age 22–24) participated in this study. Three confederates were member of the local student drama society in Maastricht and two were clinical psychology master students. They were trained for 4 h to follow a scripted protocol and to engage in consistent reserved but friendly behaviour during the waiting room and getting-acquainted task. For the waiting room they were instructed to seek eye-contact twice with the participant and then ask the question ‘Have you been participating in research before?’ After this question they were trained to leave the burden of the conversation with the participant. For the getting-acquainted task they were also instructed to leave the burden of the conversation with the participant but here they were allowed to take the initiative if the participant was silent for 7 s. Moreover, they were trained to constrain their answers to three pieces of information per answer. These instructions for the getting-acquainted task were based on prior studies by Boone et al. (1999); Öst et al. (1981); Voncken et al. (2008, 2010).

To estimate the integrity of the confederates, their behaviour was rated by the video-raters on friendliness, leaving the burden of the conversation with the participant, constraining their answers to three pieces of information and taking the initiative only after 7 s of silence by means of a 9 point Likert scale. Overall the confederates behaved friendly (waiting room M = 7.1, SD = 1.1; getting-acquainted task M = 7.4; SD = 0.9), left the burden of the conversation with the participant (waiting room M = 7.1, SD = 1.1; getting-acquainted task M = 7.4; SD = 0.9), constrained their answers to three pieces of information (waiting room M = 8.1, SD = 0.9; getting-acquainted task M = 7.4; SD = 0.8) and took the initiative only after 7s of silence (waiting room M = 8.4, SD = 0.6; getting-acquainted task M = 7.8; SD = 1.3). Independent *t* tests indicated that the confederates did not behave differently toward the low and high SAs (waiting room all *p*’s > 0.10; getting-acquainted task all *p*’s > 0.10). Thus it can be concluded that the integrity of the confederates was good.

### Video-Raters

Three video-raters, clinical psychology master students (aged 22–24), one male and two females, of the Maastricht University rated the first impression, likeability, the self-disclosure behaviour of the participants and the integrity of the confederates. They were trained for 4 h. Both confederates and video-raters were blind for the anxiety level of the participants.

### Procedure

Participants that were selected, based on their ratings from the pre-screening, received an email or a phone call with the invitation to participate in this study. Here they were informed that the assessment consisted of filling out questionnaires, conducting a computer task (reported in Voncken et al. 2011) and a social task. Participants that responded to this invitation were scheduled for assessment. On arrival in the laboratory, the experimenter showed the participant the area where the social task would be conducted. Here two chairs and a camera were placed. The experimenter took place in one chair and while pointing to the camera said:

This is the camera. After a computer task you will need to take place at this chair. Please take a seat now. The other person will sit in my chair. The person with whom you will conduct a conversation will also be part of the computer task. During the social task it is important that you do not move the chair, otherwise it will not be taped well. The purpose of the conversation is to get to know each other. What is important is that you are the one that starts the conversation and keeps it going. The camera will tape the conversation. Video observers will, later on, judge you on your social skills.

After this instruction the participants filled out a set of questionnaires, including the SIAS, and the computer task.

After filling out the questionnaires, the participant was asked to take place in the waiting room while the examiner was supposed to prepare the setting for the social task. In the waiting room the confederate was already seated. A short role-played conversation between the examiner and confederate made the participant believe that this was not the person that would participate in the upcoming social task and gave the confederate the opportunity to note his first impression of the participant. Moreover, the conversation made the participant believe that the confederate with whom the participant was supposed to have the 5 min social task, was late. The participant was told that her actual conversation partner could be there any minute. Thereupon the examiner left the room and closed the door for exactly 5 min. In this period the confederate was instructed for two times to try to make eye contact with the participant and asked the participant the question: “Have you been participating in research before?” This gave the participant the opportunity to start a conversation. (See also confederates in the method section). After 5 min the examiner came in the room to report that their conversation partner was not able to make the appointment, therefore the seated confederate was asked to participate in the social task. The confederate, of course, agreed with this request. Before the getting-acquainted task both the participant and confederate were asked to fill out the DFI. After approximately 5 min the conversation, which was taped, was cut short. The participant as well as the confederate was once more asked to fill out the DFI. Then the participant was debriefed and asked for permission to use the video material taped in the waiting room. All participants agreed.

### Data Construction of Residual Changes Scores of Likeability

To get an estimate the increase or decrease in likeability from each assessment moment (first impression, after waiting room and after getting-acquainted task) residual change scores of likeability were calculated based on de Vaus (2008, p. 156). For the waiting room we calculated the unstandardized predicted value of the DFI after the waiting room with first impression as predictor of this DFI score. We then subtracted this predicted score from the actual DFI rating after the waiting room. The higher this residual change score, the more the participant was liked than would be expected based on the first impression rating. For the getting-acquainted task we used the same procedure. Here the unstandardized predicted value of the DFI after the getting-acquainted task was predicted with the DFI rating after the waiting room and this predicted score was subtracted from the actual DFI rating after the getting-acquainted task.

Reason to use residual change scores and not differential scores, is that our first impression rating scale was a different scale than the DFI rating scale, which makes it impossible to calculate a raw change score (e.g. DFI after the waiting room minus the first impression) during the waiting room. Moreover, the problem with using raw change scores (e.g. DFI after getting-acquainted task minus DFI after waiting room) is that the amount of change is dependent on initial scores (de Vaus 2008). For instance, a participant with an initial high score will have less room for improvement than a participant with a low likeability score.

## Results

### Likeability Ratings

Means and standard deviations of the first impression and DFI are displayed in Table 3. The high SAs received a lower first impression than the low SAs, t (47) = 3.1, *p* = 0.003, d = 0.84. To investigate the likeability ratings for each group in the waiting room and in the getting-acquainted task, we conducted a repeated measures analyses with task (waiting room vs. getting-acquainted task) as a within subjects variable and group (high vs. low SAs) as a between subjects variable. This analyses showed a main effect of group, F (1, 47) = 13.9, *p* = 0.001, *η*_*p*_^*2*^ = 0.23; no main effect of time F (1, 47) = 0.79, *p* = 0.786, *η*_*p*_^*2*^ = 0.02; and an interaction between time and group F (1, 47) = 6.63, *p* = 0.013, *η*_*p*_^*2*^ = 0.12. Paired samples t-test to follow up this interaction showed that the likeability ratings of the low SAs did not change over time, t (24) = 1.1, *p* = 0.281. The high SAs, however, did improve significantly from the waiting room to the getting acquainted task, t (23) = −2.7, *p* = 0.012. Nevertheless, the high SAs received a lower likeability rating than the low SAs in both types of tasks t_*waiting room*_ (47) = 4.4, *p* < 0.001; t_*getting*-*acquainted task*_ (47) = 2.2, *p* = 0.031.

**Table 3:** Means and standard deviations of the first impression ratings, the DFI scores, residual change scores and self-disclosure behaviour

	High SAs n = 24 mean (SD)	Low SAs n = 25 mean (SD)
First impression	6.3 (0.8)	6.9 (0.6)
Likeability		
DFI after the waiting room	2.8 (0.8)	3.7 (0.7)
DFI after the getting-acquainted task	3.1 (0.7)	3.6 (0.7)
Residual change score		
Waiting room^a^	−0.21 (0.5)	0.20 (0.6)
Getting-acquainted task^b^	0.03 (0.5)	−0.03 (0.6)
Self-disclosure behaviour rating scale		
Waiting room	3.6 (2.1)	5.6 (1.7)
Getting-acquainted task	5.7 (1.0)	6.6 (0.9)

^a^DFI rating after the waiting room minus the unstandardized predicted value of the DFI after the waiting room with first impression as predictor

^b^DFI rating after the getting-acquainted task minus the unstandardized predicted value of the DFI after the getting-acquainted task with DFI rating after the waiting room as predictor

### Self-Disclosure Behaviour

First, to investigate the self-disclosure behaviour for each group in the waiting room and in the getting-acquainted task, we conducted a repeated measures analyses with task (waiting room vs. getting-acquainted task) as a within subjects variable and group (high vs. low SAs) as a between subjects variable. Means and standard deviations of the self-disclosure behaviour are displayed in Table 3. A main effect of task appeared, F (1, 42) = 32.4, *p* < 0.001, *η*_*p*_^*2*^ = 0.44, indicating that more self-disclosure behaviour was observed in the getting-acquainted task than in the waiting room. Moreover, a main effect of group appeared, F (1, 42) = 14.9, *p* < 0.001, *η*_*p*_^*2*^ = 0.26, indicating that the high SAs showed less self-disclosure behaviour than the low SAs. Furthermore, a borderline interaction effect between task and group was present, F (1, 42) = 3.7, *p* = 0.061, *η*_*p*_^*2*^ = 0.08. A *t* test was conducted to test the difference (getting acquainted - waiting room) in self-disclosure behaviour between both groups. This specified that the high SAs showed a slightly greater increase in self-disclosure behaviour than the low SAs, t (42) = 1.9, *p* = 0.061, indicating that the difference between the groups was slightly more pronounced in the waiting room compared to the getting-acquainted task.

### Prediction of Change in Likeability by Group and Self-Disclosure Behaviour

To examine the simple main effects, two *t* tests were conducted to test the difference between high and low SA in the change of likeability for both tasks (see Table 3 for the mean residual change scores). These showed a significant effect for the residual change score of the waiting room, t (47) = 2.55, *p* = 0.014, d = 0.74, but not of the getting-acquainted task, t (47)d = −0.33, *p* = 0.741, d = 0.11. Second, pearsons-r correlations showed that there is a positive relation between self-disclosure behaviour and the residual change scores in both tasks, r_*waiting room*_ = 0.60, *p* < 0.001; r_*getting*-*acquainted task*_ = 0.60, *p* < 0.001). Two regression analyses were conducted to test if group, the self-disclosure behaviour and the interaction between these two variables can explain the residual change scores of likeability during (1) the waiting room and (2) during the getting-acquainted task. Regression coefficients are displayed in Table 4. Both regression analyses showed a significant effect of self-disclosure, but no main effect of group and no interaction between group and self-disclosure. Thus, independent of social anxiety, the more self-disclosure participants displayed, the more they increased in likeability.

**Table 4:** Standardized regression coefficients of regression analysis to predict the residual change of likeability during the waiting room (i.e., from first impression to the DFI after the waiting room) and during the getting-acquainted task (i.e., from DFI after the waiting room to after the getting-acquainted task)

	*Β*	*β*	*R* ^2^
Waiting room			0.37
Group	0.21	0.21	
Self-disclosure behaviour	0.17*	0.71	
Interaction between group and self-disclosure behaviour	−0.04	−0.19	
Social task			0.44
Group	1.0	0.97	
Self-disclosure behaviour	0.44**	0.84	
Interaction between group and self-disclosure behaviour	−0.11	−0.63	

*p < 0.005. ** *p* < 0.001

## Discussion

In this study we revealed that already in the very first seconds of contact high SAs elicit a more negative impression than low SAs. Furthermore, also after subsequent interactions high SAs receive a lower judgment concerning their likeability than low SAs. However, this study also demonstrates that SAs likeability ratings do improve: high SAs were rated more positive after the second social task than after the first waiting room task. Self-disclosure behaviour might help to change this negative impression. In both social interactions the level of self-disclosure behaviour was the strongest predictor for the increase of likeability. The absence of an interaction between group and self-disclosure suggests that this is positive effect of self-disclosure exists for both groups. This suggests that high SAs can make up for their negative first impression by increasing their self-disclosure behaviour. However, this study also showed that high SAs used less self-disclosure behaviour than low SAs during both social interactions, a difference that was most pronounced in the waiting room situation. This may implicate that it is problematic for high SAs to improve their self-disclosure behaviour.

This study adds to the body of studies demonstrating the social anxiety—social rejection relationship (Alden and Wallace 1995; Creed and Funder 1998; Heerey and Kring 2007; Meleshko and Alden 1993; Pilkonis 1977; Voncken et al. 2008, 2010) and shows, again, that there is a core of truth in the negative beliefs SAs hold about being less likeable than others. This study was, to our knowledge, the first study to elucidate that high SAs in the very first seconds of social contact elicit a negative impression. Thus the social anxiety-social rejection relation is already established very early in social contact. What exactly elicits this negative impression of SAs in others remains to be investigated. Safety behaviours such a dressing inconspicuous, frowning or a posture with the message of social withdrawal might play an important role here.

This negative first impression of high SAs sustained and even got worse during the natural, unstructured waiting room situation. That is, a significant main effect of group showed that, compared to low SAs, high SAs received a more negative likeability rating after the waiting room than could be predicted on basis of their very first impression. In other words, interacting with others in a natural setting does make things worse, as high SAs tend to believe and as one might expect from the disturbing effect of safety behaviours (see McManus et al. 2008 and Taylor and Alden 2011). In contrast the low SAs received a more positive likeability rating in this respect. This is in line with Wilder and Thompson (1980) who demonstrated in healthy participants that people form more favourable views toward whomever they spent time with.

This study also demonstrates that although high SAs elicit a more negative impression, self-disclosure behaviour might help to repair this impression. That is, the more self-disclosure behaviour the participants showed the more likeable they were rated after the waiting room than what would be predicted on basis of their first impression; even to the extent that the level of self-disclosure behaviour was a stronger predictor here than the level of social anxiety (i.e., the group effect). This fits with findings of Papsdorf and Alden (1998) and Taylor and Alden (2011). These studies showed that during a social interaction positive interpersonal outcomes were mainly predicted by the approach or open behaviour the participants displayed and not by their visible anxiety symptoms.

Furthermore, in the waiting room as well as during the getting-acquainted task the more self-disclosure behaviour the participants showed the more likeable they were rated after the getting-acquainted task than what would be predicted on basis of their rating right after the waiting room. Thus, it seems that socially anxious individuals can to overcome this first hurdle of their first negative impression if they show consistent self-disclosure behaviour they are eventually perceived as likeable individuals. This may well reflect patients’ reports such as: ‘Friends told me that they initially didn’t like me that much but started to appreciate me more and more when they got to know me better’.

Although the negative first impression of high SAs is open for improvement with the use of self-disclosure behaviour, the question is whether high SAs are able to increase their self-disclosure behaviour. Our results clearly show that the SAs group has shortcomings in this type of behaviour. This shortcoming was more pronounced in the waiting room situation than in the getting-acquainted task. As the waiting room situation was a more unstructured, natural, social interaction it generalizes best to real life situations compared to the getting-acquainted task. This indicates the under more natural circumstances behaviour problems of SAs could well be worse than in the structured settings as a getting-acquainted task or a speech situation as studied in many previous studies before (Baker and Edelmann 2002; Beidel et al. 1985; Bögels et al. 2002; Daly et al. 1978; Fydrich et al. 1998; Lewin et al. 1996; Stopa and Clark 1993; Thompson and Rapee 2002; Twentyman and McFall 1975; Voncken and Bögels 2008). Encouraging is, on the other hand, that in such a structured getting-acquainted task, socially anxious individuals were able to show more self-disclosure behaviour than in the unstructured waiting room situation. Moreover, Alden and Wallace (1995) demonstrated that SAD patients showed more skilful behaviour when interacting with a responsive partner versus a non-responsive partner. This indicates that high SAs are flexible in their behaviour patterns and, therefore, might be able to learn to apply self-disclosure behaviour and improve the social outcomes of their interactions. This corresponds well with a new addition to current treatment regimens developed by Alden and Taylor (2011) for patients with generalised SAD. In their treatment, patients, instead of only experimenting with dropping of safety behaviours (based on the work of Clark 2005; Clark et al. 2006), also extensively experiment with increasing their social approach and reciprocal self-disclosure behaviour. This regimen was accepted well by the patients, resulted in high effect-sizes in improving social anxiety and has the potential to repair the problematic relationships these patients have. However, it was not investigated whether they truly showed more social approach behaviour after treatment and had more positive outcomes in social interactions.

With regard to the assessment of self-disclosure behaviour, we found that negative open behaviour appeared infrequent. This seems to reflect that in first social encounters it is not custom to share negative emotions with others (Altman and Taylor 1973). However, for the development of meaningful and intimate friendships such openness about negative emotions is thought to be of high importance (Altman and Taylor 1973). A number of studies have demonstrated that SAs avoid emotional expression and emotional closeness in intimate social relations (Davila and Beck 2002; Grant et al. 2007; Sparrevohn and Rapee 2009; Wenzel 2002). So, although in this study the negative self-disclosure scale was abundant, it might be important to investigate this type of behaviour in the development of intimate social relations.

Clinically, therapists need to keep in mind that there is some validity in the beliefs SAD patients that they are less liked than others even to the extent that their appearance already elicits more social rejection. We did not get insight in what exactly elicited this very first negative impression. But in treatment one might try to address safety behaviours that affect such a first impression, such as posture, frowning, or wearing inconspicuous clothing or hair dress. Moreover, this study appears to indicate that helping patients to show more self-disclosure behaviour facilitates repairing the reduced likeability of SAs. The treatment regimen developed by Alden and Taylor (2011) can be of value here. It seems important to target all three self-disclosure behaviours investigated in this study as all were related to likeability. Some safety behaviours in SAD may affect these self-disclosure behaviours. For instance, some patients refrain from personal self-disclosures in order to reduce the probability that they will be rejected, or tend to overload their interaction partner with a barrage of questions in order to reduce their own speech time or are so busy focussing on questions to ask that they are not able to genuine listen to their interaction partner. Addressing such safety behaviours may help to improve self-disclosure behaviour in SAD. Last, it is important to note that, although the social anxiety—social rejection relationship is demonstrated in various studies, SAD patients, do overestimate the negative evaluation by others (among others see Voncken and Bögels 2008). Moreover, Voncken et al. (2010) showed that negative beliefs play an important role in the social anxiety—social rejection relationship. Therefore, addressing negative beliefs with current cognitive techniques is of value in treatment of SAD.

This study suffered from several limitations. First, we used an analogue population of women who were mainly psychology students. The social anxiety—social rejection relationship has been demonstrated also in male and female SAD patients and healthy control participants with various education profiles (Voncken et al. 2008). Nevertheless it is important the replicate the current findings in a mixed-sex group of clinically anxious individuals. Second, we used a laboratory social interaction setting. Although, the waiting room situation reflected a more natural social interaction, our getting-acquainted task remains artificial. That is, the participants were instructed to get to know their interaction partner. Therefore, the question remains whether in real life, without such clear instructions, high SAs would indeed show more self-disclosure behaviour. Furthermore, we did not manipulate self-disclosure behaviour. Therefore, the study does not allow causal inferences about the effect of self-disclosure on liking. An important next step would be to determine whether an increase in self-disclosure behaviour would indeed lead to greater likeability of SA individuals. Despite these limitations, the current study adds to the evidence of the social anxiety—social rejection relationship and demonstrated that high SAs get less positive judgments already after a few seconds. However, they do get a second change after being disliked at first sight and the increase of self-disclosure behaviour seems to play an important role here. Research is needed to see whether SAs are able to increase their self-disclosure behaviour and whether this repairs their negative social interaction cycles.
